# Zeptomole Electrochemical Detection of Metallothioneins

**DOI:** 10.1371/journal.pone.0011441

**Published:** 2010-07-06

**Authors:** Vojtech Adam, Jitka Petrlova, Joseph Wang, Tomas Eckschlager, Libuse Trnkova, Rene Kizek

**Affiliations:** 1 Department of Chemistry and Biochemistry, Faculty of Agronomy, Mendel University in Brno, Brno, Czech Republic; 2 Department of Nanoengineering, University California San Diego, La Jolla, California, United States of America; 3 Department of Paediatric Haematology and Oncology, 2nd Faculty of Medicine, Charles University, Prague, Czech Republic; 4 Department of Chemistry, Faculty of Science, Masaryk University, Brno, Czech Republic; Instituto de Tecnologia Química e Biológica, Portugal

## Abstract

**Background:**

Thiol-rich peptides and proteins possess a large number of biological activities and may serve as markers for numerous health problems including cancer. Metallothionein (MT), a small molecular mass protein rich in cysteine, may be considered as one of the promising tumour markers. The aim of this paper was to employ chronopotentiometric stripping analysis (CPSA) for highly sensitive detection of MT.

**Methodology/Principal Findings:**

In this study, we used adsorptive transfer stripping technique coupled with CPSA for detection of cysteine, glutathione oxidized and reduced, phytochelatin, bovine serum albumin, and metallothionein. Under the optimal conditions, we were able to estimate detection limits down to tens of fg per ml. Further, this method was applied to detect metallothioneins in blood serum obtained from patients with breast cancer and in neuroblastoma cells resistant and sensitive to cisplatin in order to show the possible role of metallothioneins in carcinogenesis. It was found that MT level in blood serum was almost twice higher as compared to the level determined in healthy individuals.

**Conclusions/Significance:**

This paper brings unique results on the application of ultra-sensitive electroanalytical method for metallothionein detection. The detection limit and other analytical parameters are the best among the parameters of other techniques. In spite of the fact that the paper is mainly focused on metallothionein, it is worth mentioning that successful detection of other biologically important molecules is possible by this method. Coupling of this method with simple isolation methods such as antibody-modified paramagnetic particles may be implemented to lab–on-chip instrument.

## Introduction

According to World Health Organization statistics, cancer has taken up the first position among the killer diseases in the population of the developed countries. Due to this fact, the significance of the tumour diagnostics is prodigious and it is focused on various tumour markers. Numerous different oncomarkers (e.g. CA-15, PSA) were set up in the oncological practice and many alternative oncomarkers are intensively investigated [1], [2], [3], [4]. It seems that the low-molecular mass compounds including thiols may play a crucial role in this area. Metallothionein (MT) can be included in this group of highly interesting thiol-rich proteins. It was discovered in 1957, when Margoshes and Valee isolated MT from horse kidneys [5]. The main biological function of MT is the homeostatic control and detoxification of heavy metals (zinc, copper, cadmium). Often discussed question is the ability of MT to transport the ions of heavy metals to the apoenzymes and scavenging of the reactive oxygen radicals [6]. Several papers investigate by different methods and discuss the detection of metallothioneins [7], [8], [9], [10]. Capillary electrophoresis, liquid chromatography mass spectrometry, inductive coupled plasma mass spectrometry, immunoassays and electrochemistry was used in abovementioned works and the results from these papers have been reviewed [11], [12]. The aim of this paper was to utilize chronopotentiometric stripping analysis (CPSA) for highly sensitive detection of MT.

## Results and Discussion

**Figure 1:**
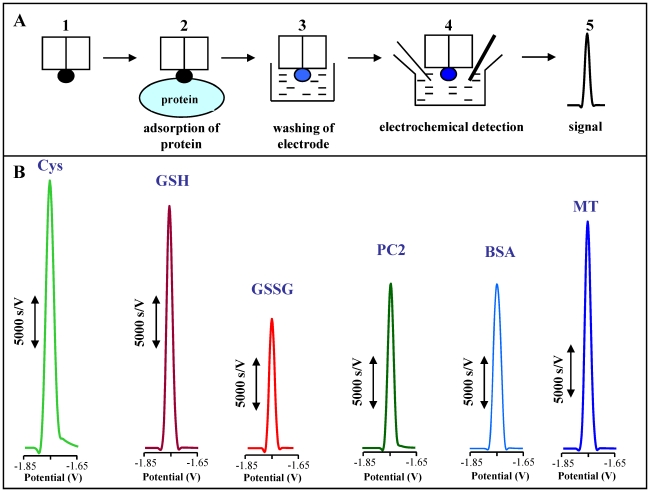
Adsorptive transfer technique. The adsorptive transfer technique is based on the sample accumulation onto the working electrode surface and consequently on the electrode washing and measurement (A). CPSA scans of Cys, GSH, GSSG, PC2, BSA and MT in concentration of 100 nM (B). The supporting electrolyte was composed of 0.1 M H_3_BO_3_+0.05 M Na_2_B_4_O_7_. AdTS CPSA parameters were as follows: starting potential 0 V, ending potential –1.85 V, temperature 20°C, time of accumulation 120 s; no reducing agent was added.

The development of adsorptive transfer technique (AdTS) markedly promoted the use of electrochemical techniques for the detection of the adsorptive substances which are biologically active compounds, such as DNA or proteins. Our group was previously interested in MT determination by using CPSA in the combination with AdTS. We found that the components of the supporting electrolyte are participating in catalytic process itself as well as in MT protonization [13], [14], [15]. The principle of AdTS is shown in Fig. 1A.

Thiols (cysteine, glutathione oxidized and reduced, phytochelatin, and metallothioneins) and bovine serum albumin were analysed by using AdTS CPSA to demonstrate the applicability of these methods. Well-developed catalytic signals of individual thiols (Fig. 1B) were obtained. These signals linearly depended on the thiols concentrations (Table 1). The estimated detection limits of analysed samples were in tens fg of ml. The relative standard deviation (RSD) varied from 4.2 to 8.9% according to the molecular size of individual thiols and their -SH groups accessibility to the surface of the working electrode. It is evident that the ultrahigh sensitivity with the combination of the analysis of microlitre sample volume makes from this analytical technique one of the most sensitive tools for the analysis of thiols.

**Table 1:** Detection limits of thiols (*n* = 5) using AdTS CPSA method.

Concentration of thiol^a^ (pg/ml)	Regression equation	R^2^	LOD b (fg/ml; zmol*)	LOQ c (fg/ml; zmol*)
Cysteine (0.2–10)	y = 50.656x+58.569	0.9968	70; 3,000	230; 10,000
Glutathione (GSH) (0.5–10)	y = 8.6786x+29.564	0.9973	20; 300	70; 1,000
Glutathione (GSSG) (1–10)	y = 8.6489x+29.982	0.9982	40; 300	130; 1,000
Phytochelatin (PC2) (0.1–10)	y = 54.114x+11.576	0.9956	60; 600	200; 2,000
Bovine serum albumin (0.5–10)	y = 11.829x+20.185	0.9912	30; 2	100; 7
Metallothionein (0.1–10)	y = 6.9251x+40.651	0.9980	10; 11	30; 36

a The linear section dependence of the studied thiol concentration.

b The limit of Detection (3 S/N).

c The limit of Quantification (10 S/N).

*Per drop sample (5 µl).

### Reducing agent

**Figure 2:**
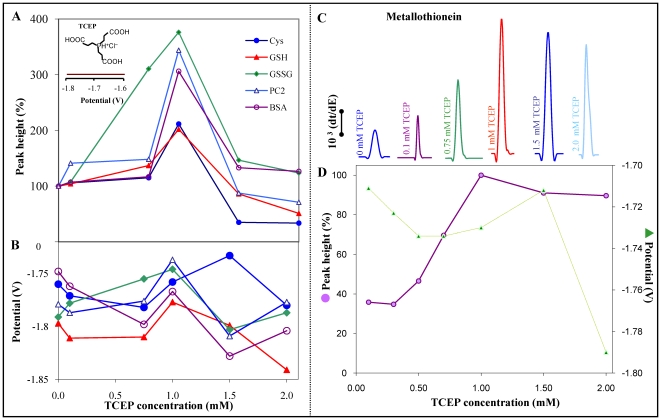
The effect of tris(2-carboxyethyl)phosphine (TCEP). Dependences of peak H height of Cys, GSH, GSSG, PC2, BSA (A) and its potential (B) on the different TCEP concentration; the picture in inset: chemical structure of TCEP. The influence of TCEP on CPSA signals of 40 fmol MT (5 µl, C). Dependences of peak H height and potential of 40 fmol MT (5 µl) on different TCEP concentration (D). The peak height 55 000 s/V corresponds to 100% (B).

Oxidation of proteins -SH groups can influence their respective analytical determination. For the purpose of sensitive detection of selected thiol, its reduction may be advantageously used. Primarily, we were interested in the influence of the tris(2-carboxyethyl)phosphine (TCEP) reducing agent on the AdTS CPSA signals of all studied thiols. It was found that 1 mM TCEP does not provide any signal at the negative potentials about −1.7 V (in inset in Fig. 2A). The changes of the peak H with varying phosphine concentration were studied (Fig. 2A,B). Signals of examined thiols quickly increased in accordance with phosphine concentration; maximum was reached at the concentration of 1 mM. Subsequent increasing of phosphine concentration led to the signal height reduction which was probably caused by changes of electrochemical processes on the working electrode surface. Figure 2A shows the significant change of GSSG reduction (100 nM) by GSH forming. The arising GSH signal corresponds to the signal of 200 nM GSH. The changes in potentials of signals are shown in Fig. 2B. The phosphine concentrations higher than 1 mM lead to the distinct shift of the signals to negative potentials.

As reported previously, the metallothionein level may be related to the malignant disease and its development, progress or expansion [6], [16], [17], [18], [19], [20]. Because of this fact, our experimental work was mainly focused on MT research. Initially, we reduced 500 fmol MT by 1 mM TCEP and determined increase of MT signal for more than 50% (Fig. 2C). Further, we were interested in TCEP concentration, exactly in the most suitable TCEP concentration for MT determination. Thus, we stepwise added different TCEP concentrations (0.1–1.5 mM) to MT. The observed MT signal moved with increasing TCEP concentration towards more negative potentials and it was increased to the concentration of 1 mM TCEP. After reaching of the peak H maximum, it remained practically unchanged (Fig. 2D).

### Stripping Current and Temperature

**Figure 3:**
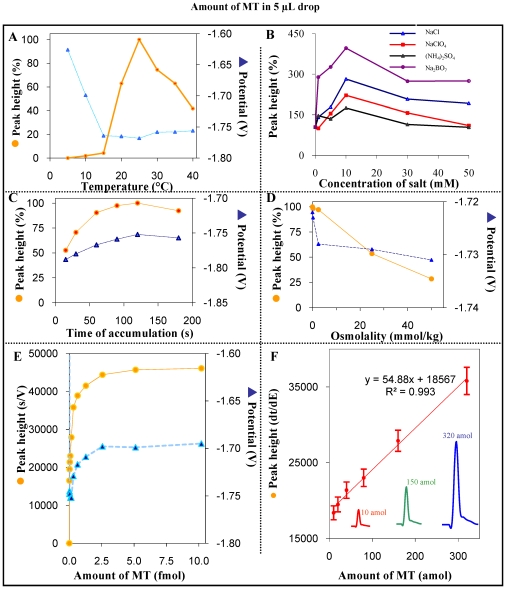
The affecting of peak H by various experimental conditions. The dependence of peak H on the temperature (A), different ionic strength (B), time of accumulation (C), osmolality (D) and concentration varying from 0 to 10 fmol (E) and from 0 to 320 amol (F). The concentration of MT was 5 fmol in 5 µl drop of sample. The signal height 40 000 (5 fmol) and 60 000 (500 fmol) s/V corresponds to 100%.

During protein detection, the height of peak H depends on stripping current (I_str_). The most proper tripping current for MT detection was apparently determined as I_str_ = 1 µA. When higher I_str_ values were used, the signal of MT was expressively reduced. Stripping current lower than 1 µA extended the measurement times to more than five minutes. The effect of temperature on the catalytic signal in two MT concentrations (5 fmol and 500 fmol for 5 µl drop of sample) was also determined. MT was adsorbed onto the HMDE surface for 120 s at room temperature (22°C) and then it was washed in the solution of the supporting electrolyte. HMDE electrode modified in this manner was transferred into the supporting electrolyte solution of the strictly defined temperature (5, 10, 15, 20, 25, 30, 35, and 40°C). Low temperatures led to the MT catalytic signal reduction. The MT signal increased rapidly depending upthe increasing temperature while shifting of signal to more negative values (about 80 mV). The highest signal was observed by using both the studied concentrations at 25°C (Fig. 3A). According to our observations, the catalytic signal (peak H), respectively its height and position, depends on temperature. It is interesting that the process of H peak catalysis in the presence of cobalt ions (Brdicka reaction) differs significantly when compared with the above-mentioned results. The highest signal in this case was determined at 5°C [21].

### Interferences

The presence of various ions (anions as well as cations) may significantly affects the results of the real samples. In this study, the influence of four different ions (NaCl, NaClO_4_, (NH_4_)_2_SO_4_ and Na_2_BO_7_) in the solution containing MT was tested. All tested ions in the monitored concentrations had the positive effect on the catalytic MT signal. Its maximum was observed at concentration of 10 mM in the case of all tested ions. The most distinct signal was determined in the presence of sodium tetraborate (Fig. 3B). In the presence of the ions at high concentrations, the MT catalytic signal declined (Fig. 3D). Figure 3C shows the influence of the MT accumulation time (5 fmol to 5 µl drop of sample) on MT electrochemical response. The response increased up to 120 s. By comparison of two MT concentrations (5 and 500 fmol to 5 µl drop of sample), signal increasing was more evident in the second case - 500 fmol of MT to 5 µl drop of sample. After reaching the signal maximum, it declined because of the electrode surface saturation. In comparison with the concentration of 5 fmol MT to 5 µl drop of sample (900 fM), changes in signal height were not observable (time of accumulation 120 s) (Fig. 3C). The observed MT signals stepwise shifted to more positive potentials in dependence on the increasing time of accumulation. As it is obvious from the presented results, the most suitable time of accumulation is 120 s.

### Detection limit

Characteristic CPSA concentration dependence was obtained after analysis of very low concentrations of reduced MT (from 100 nM) using 120 s as the time of accumulation and temperature-controlled supporting electrolyte 25°C (Fig. 3E). This dependence was typically Langmuir-isotherm shaped. It was possible to divide obtained concentration dependence into two separate linear sections: i) 0.6–10 fmol in 5 µl drop (y = 1954.5x+38600; R^2^ = 0.9926; not shown) with relative standard deviation 4.8% (n = 10) and ii) 10–320 amol in 5 µl drop (y = 54.884x+18567; R^2^ = 0.9937; Fig. 3F). At these very low MT concentrations (10, 150 and 320 amol in 5 µl drop), the observed signals were very well developed. The analysis itself took no more than 5 min. Relative standard deviation was 6.5% (n = 10). Due to our newly modified electrochemical technique, 3 S/N (signal/noise, according to Long and Winefordner [22]) detection limit of metallothionein in the supporting electrolyte was determined as 11 zmol in 5 µl drop. Besides CPSA, other voltammetric methods have been used for determination of MT. The most sensitive ones is differential pulse voltammetry Brdicka reaction [23]. Detection limit estimated by authors was 500 zmol in 500 nl. In spite of the fact that the method is very sensitive, CPSA detection limit mentioned above is more than for hundred times lower. However, we attempted to utilize the main improvement from the mentioned paper, cooled parafilm with a drop of sample, for our purposes. We were able to reduce drop volume down to 500 nl without negative effects such as increasing relative standard deviation on a measurement. Moreover, we were able to detect 1 zmol in 500 nl drop.

### Analysis of human blood serum samples

**Figure 4:**
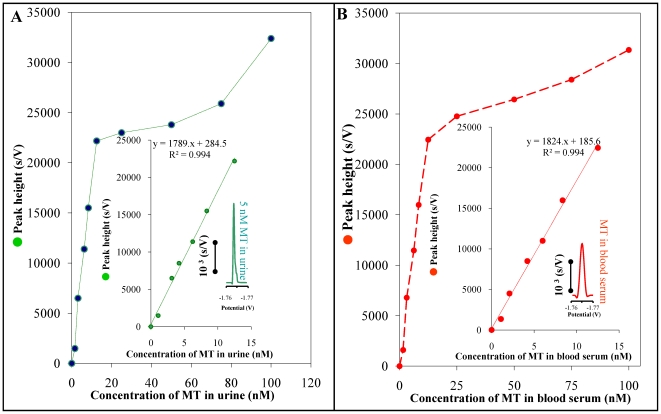
Concentration dependences. The dependence of the peak H height on MT addition to the sample of human urine (A) and blood serum (B) of healthy people. In the inset pictures, the linear sections of studied dependences are shown. Chronopotentiogram demonstrates the signal 5 nM of MT after the addition into the human urine and signal of MT in blood serum.

The highly sensitive technique AdTS CPSA for MT determination, which has been developed and described by our group, was applied to MT determination in human urine and blood serum of the healthy individuals. Firstly, our attempt was focused on the determination of MT in urine using AdTS CPSA technique. We determined that high ionic strength (osmolality) negatively influences peak H (Fig. 3D). In the case of osmolality higher than 50 mmol/kg, observed signal was reduced for more than 50%. Due to this fact, it was necessary to dilute properly the studied urine sample because of decrease of osmolality and sensitivity preservation. Different amounts of MT were added to the sample of urine (0.25 mmol/kg). The observed dependence is shown in Fig. 4A. Rapid signal increase (up to the MT concentration of 10 nM) is obvious. Higher MT concentrations did not results in the proportional increasing of the signal height. At low concentrations, it was possible to establish the linear dependence with equation y = 1789.4x+284.51 and R^2^ = 0.9949. The relative standard deviation was 4.9% (n = 5), in inset in Figure 4A. It was found that MT level is changed in the case of heavy liver diseases, such as hepatocellular carcinoma, chronic hepatitis, and liver cirrhosis. We decided to use AdTS CPSA technique for MT determination in human blood serum. The sample was heat-denatured and centrifuged; after centrifugation, supernatant was used for MT analysis. The sample was thousand times diluted and consequently analysed in 5 µl drop. In the case of more concentrated sample, the peak height was reduced for about 25% (in comparison with 10 times diluted sample), probably due to very strong adsorption onto HMDE surface. Into the sample of blood serum, the different MT amounts were added (Fig. 4B). In the MT concentrations of 0–15 nM, strictly linear dependence of standard addition on increasing MT concentration (y = 1824.2x+185.65, R^2^ = 0.9949) was determined. The relative standard deviation was about 4.7%. In our samples of human blood serum, average MT concentration was determined as 0.75±0.02 µM (n = 5). The inset in Figure 4B shows the chronopotentiogram corresponding to 0.8 µM MT concentration in blood serum sample.

**Figure 5:**
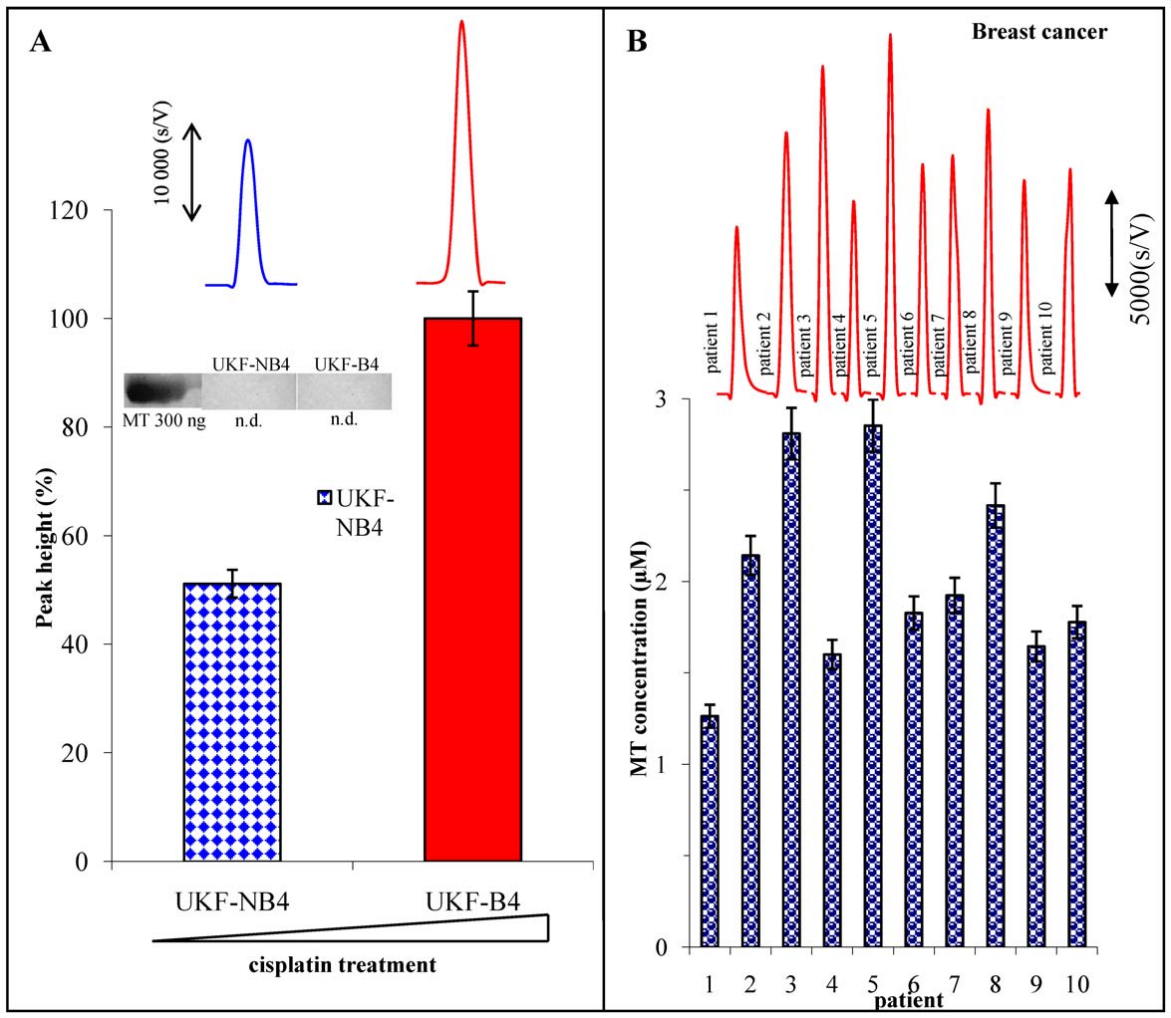
Cancer samples. The total amount of MT analysed in neuroblastoma cells sensitive (UKF-NB4) and resistant (UKF-B4) to cisplatin. SDS-PAGE band in MT position (300 ng for the control) and chronopotentiograms are displayed as the inset pictures (A). The MT chronopotentiograms of the human blood serum of the patients with the malignant breast tumour and MT amount of the individual patients (B).

Antitumor drug resistance, such as to cisplatin (cisplatinum or cis-diamminedichloroplatinum(II) (CDDP)), represents very consequential antitumor therapy failure. It is supposed that metallothionein plays very important role in this area [6], [19]. Because of this fact, cisplatin-sensitive and cisplatin-resistant tumour neuroblastoma cell lines cultivated in the presence of 1 mM cisplatin were analysed. Using a common SDS PAGE electrophoresis, MT was not determined in these tumour cell lines (Fig. 5A). Electrochemical analysis demonstrated the presence of MT in investigated samples. Signal increased for more than 50% was determined in the samples of cisplatin-resistant cell line in comparison with cisplatin-sensitive cell line. AdTS CPSA may constitute very important tool in MT analysis of the patients with malignant tumours for the clinical diagnosis.

Influence of the malignant tumour disease to the MT signal in the blood serums of the patients with the malignant breast tumours was also determined. Figure 5B shows the characteristic chronopotentiometric scans of the individual patients with the breast tumour. The MT level in these samples ranged from 1.3 to 2.8 µM. Each sample was three times analysed; relative standard deviation was 5.5%. The MT level of the healthy people was established as 0.75 µM, thus it means that patients with malignant disease have increased MT level in the blood serum for more than 170% on average.

## Materials and Methods

### Chemicals

Rabbit liver MT, containing 5.9% Cd and 0.5% Zn, was purchased from Sigma Aldrich (MW 7143). Standard solutions of MT were prepared by diluting the stock solution (1.0 mg MT/mL) with water (Sigma Aldrich, ACS). All other chemicals were obtained from Sigma, unless noted otherwise.

### Real samples

The samples of the *human urine* were obtained from the healthy persons (the staff of the laboratory). Obtained urine was filtrated through the teflon disc filter (0.45 µm and 13 mm in diameter, Alltech Associates, Deerfield, Il, USA). For the urine osmolality determination, the fact that human urine osmolality is 250 mmol.kg^−1^ was used. *Human blood serum* was obtained from the Department of Clinical Biochemistry and Pathobiochemistry, 2^nd^ Faculty of Medicine, Charles University, Czech Republic. We analysed the samples of patients with breast cancer (n = 10, average age = 50). The research has been approved by the Independent ethics committee at Motol University Hospital, Prague, Czech Republic. The informed consent has been obtained from all participants. *The neuroblastoma tumour cell lines* were derived from the neuroblastoma metastases into the bone marrow of the patients in the relapse phase. The individual cell lines were derived by cultivation with the increasing cisplatin concentration in cultivation medium. Tumour cells were cultivated in the IMDM medium with addition of 10% foetal calf serum at 37°C, the cisplatin resistant tumour cell lines were cultivated in the above-mentioned cultivation medium with presence of cisplatin.

### Preparation of real samples

Samples were prepared by heat treatment. Briefly, the samples were kept at 99°C in a thermomixer (Eppendorf 5430, USA) for 15 min with the occasional stirring, and then cooled down to 4°C. The denatured homogenates were centrifuged at 4°C at 15 000 *g* for 30 min (Eppendorf 5402, USA). The heat treatment effectively denatures and removes the high molecular weight proteins from real samples [21].

### SDS PAGE

The electrophoresis was performed on the Biometra apparatus (Biometra, Germany). At first 15% running, then 5% stacking gel was poured; the polymerization of the running gel was carried out at room temperature for 1 h and 30 min for gel stacking. Prior to analysis, the samples were mixed with the sample buffer containing 5% 2-mercaptoethanol in the rate of 1∶1. The samples were boiled for 2 min and after denaturation, consequently loaded onto gel in volume of 20 µl. The protein ladder Precision plus protein standards from Biorad were used for the determination of molecular weight. The Coomassie blue staining of the gels was performed in accordance with Diezel, W et al. [24]. The gel was scanned and analyzed by Biolight software (Vilber-Lourmat) after the staining. The pH value was measured by using WTW inoLab Level 3 with terminal Level 3 (MultiLab Pilot; Weilheim, Germany).

### Electrochemical instrument

The electrochemical measurements were performed with AUTOLAB Analyzer (EcoChemie, Netherlands) connected to VA-Stand 663 (Metrohm, Switzerland), using a standard three-electrode cell. The working electrode was a hanging mercury drop electrode (HMDE) with a drop area of 0.4 mm^2^ using silanized glassy capillaries. The reference electrode was an Ag/AgCl/3M KCl electrode and the auxiliary electrode was a graphite electrode. GPES 4.9 software (supplied by EcoChemie) was used for the smoothing and baseline correction. For the determination of the MT content, an adsorptive transfer stripping technique in the connection with the chronopotentiometric stripping analysis were used to record the inverted time derivation of potential (*dE/dt)^−1^* as a function potential *E*. CPSA parameters were as follows: time of accumulation of 120 s, I_str_ of −1 µA. After the adsorption of MT the HMDE was washed and transferred in an electrolytic cell with 0.1 M H_3_BO_3_+0.05 M Na_2_B_4_O_7_, pH 8.6 (Sigma Aldrich, ACS) where the CPSA was performed.
